# The Diverse Functions of Non-Essential Amino Acids in Cancer

**DOI:** 10.3390/cancers11050675

**Published:** 2019-05-15

**Authors:** Bo-Hyun Choi, Jonathan L. Coloff

**Affiliations:** Department of Physiology and Biophysics, University of Illinois Cancer Center, University of Illinois at Chicago, Chicago, IL 60612, USA; bhchoi37@uic.edu

**Keywords:** aspartate, asparagine, arginine, cysteine, glutamate, glutamine, glycine, proline, serine, cancer

## Abstract

Far beyond simply being 11 of the 20 amino acids needed for protein synthesis, non-essential amino acids play numerous important roles in tumor metabolism. These diverse functions include providing precursors for the biosynthesis of macromolecules, controlling redox status and antioxidant systems, and serving as substrates for post-translational and epigenetic modifications. This functional diversity has sparked great interest in targeting non-essential amino acid metabolism for cancer therapy and has motivated the development of several therapies that are either already used in the clinic or are currently in clinical trials. In this review, we will discuss the important roles that each of the 11 non-essential amino acids play in cancer, how their metabolic pathways are linked, and how researchers are working to overcome the unique challenges of targeting non-essential amino acid metabolism for cancer therapy.

## 1. Introduction

It is now well established that tumors display different metabolic phenotypes than normal tissues [1]. The first observed and most studied metabolic phenotype of tumors is that of increased glucose uptake and glycolysis [2,3], a metabolic phenotype that is exploited in the clinic to image human tumors and metastases via ^18^flurodeoxyglucose positron emission tomography (^18^FDG-PET) [4]. In addition to glucose, there has also been a long-standing interest in understanding the unique amino acid requirements of cancer cells [5]. Indeed, like glucose, there are major differences in the uptake and secretion of several amino acids in tumors relative to normal tissues [5,6,7]. Further, it is now appreciated that amino acids, rather than glucose, account for the majority of the carbon-based biomass production in rapidly proliferating cancer cells [8]. Amino acids also contain nitrogen and have been demonstrated to be the dominant nitrogen source for hexosamines, nucleotides, and other nitrogenous compounds in rapidly proliferating cells [9,10,11]. Because of these important roles in tumor metabolism, there continues to be significant interest in targeting amino acid metabolism for cancer therapy. 

The 20 proteinogenic amino acids can be divided into two primary subgroups—essential amino acids (EAAs) and non-essential amino acids (NEAAs) [12]. This classification is based on dietary necessity, and an amino acid is deemed essential if it “…cannot be synthesized by the animal organism, out of materials ordinarily available to the cells, at a speed commensurate with the demands for normal growth [13].” In humans there are 9 essential amino acids (histidine, isoleucine, leucine, lysine, methionine, phenylalanine, threonine, tryptophan, and valine) and 11 non-essential amino acids (alanine, aspartate, asparagine, arginine, cysteine, glutamate, glutamine, glycine, proline, serine, and tyrosine). Of the 11 NEAAs, at least 6 are considered “conditionally essential” because there are physiological and/or pathological conditions where they become dietarily required, such as in the inborn error of metabolism phenylketonuria where tyrosine can no longer be synthesized and therefore needs to be consumed [14]. It is important to note that the dietary essentiality of amino acids is considered at the organismal level, as it is known that certain cell types and tissues lack the ability to synthesize or take up some NEAAs. In addition, the circulating concentrations of the 11 NEAAs in humans are highly variable, ranging from 20 µM for aspartate to 550 µM for glutamine [15]. Further, it has been observed that the concentrations of several NEAAs including glutamine, serine, and arginine can be regionally depleted within the tumor microenvironment [16,17,18]. Therefore, the availability of an amino acid to be synthesized or consumed is the result of a complex interaction between tissue-specific gene expression programs, dietary consumption, and local consumption/secretion rates. This results in an inherent complexity in NEAA metabolism that introduces unique challenges when attempting to manipulate these pathways therapeutically, especially in cancer where the levels of nutrients are highly variable [18,19].

In this review, we will focus on the diversity of the metabolic roles that NEAAs play in cancer. In addition to being 11 of the 20 amino acids needed for protein synthesis, NEAAs are important for many other aspects of tumor metabolism, including nucleotide and lipid biosynthesis, maintenance of redox homeostasis, and numerous allosteric and epigenetic regulatory mechanisms. The importance of these diverse roles has generated great interest in targeting NEAA metabolism for cancer therapy. Indeed, several NEAA-targeted therapies are already used for cancer treatment, and several others are being evaluated in clinical trials, while many more are being explored pre-clinically. The inherent complexity of NEAA metabolism has motivated the examination of numerous approaches for targeting these pathways for therapy, including inhibition of their biosynthetic pathways or key nodes of utilization, inhibition of cellular NEAA uptake, or depletion of plasma NEAA levels either through enzymatic degradation or restriction of NEAAs in the diet. In this review, we will briefly discuss each of the 11 NEAAs, how they function to support the pathology of cancer, and what strategies are currently used or are being developed for targeting NEAA metabolism for cancer therapy.

## 2. Non-Essential Amino Acids

### 2.1. Glutamine

**Figure 1:**
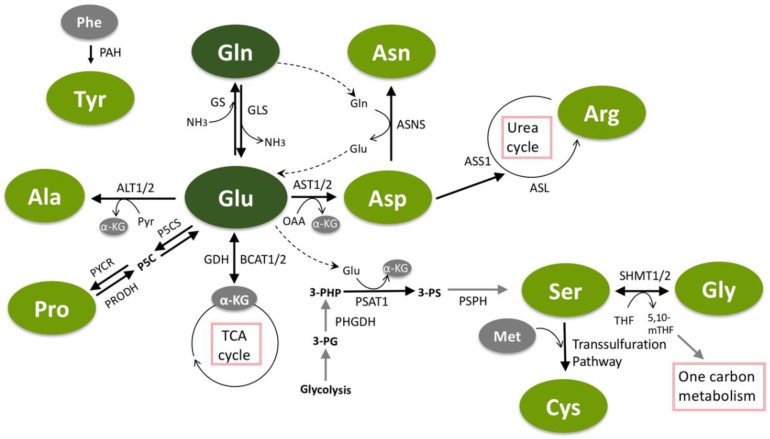
The interconnected pathways of non-essential amino acids (NEAA) metabolism. Glutamine and glutamate have a central role in non-essential amino acid metabolism, and can each be used for the synthesis of other NEAAs. Glutamate can be utilized to generate alanine, aspartate, serine and proline. Aspartate is further utilized to generate asparagine (with nitrogen from glutamine) and can be used in the urea cycle to make arginine. Serine donates methyl groups for one-carbon metabolism and makes glycine in the process. Serine can also be used in the transsulfuration pathway to generate cysteine. Tyrosine is the only NEAA not directly connected to the others, as it is separately synthesized from phenylalanine. Green circles indicate non-essential amino acids. Abbreviations: Gln = glutamine; Glu = glutamate; Phe = phenylalanine; Tyr = tyrosine; Ala = alanine; Pro = proline; Asp = aspartate; Asn = asparagine; Arg = arginine; Ser = serine; Gly = glycine; Met = methionine; Cys = cysteine; α-KG = α-ketoglutarate; ALT1/2 = alanine aminotransferase 1/2; AST1/2 = aspartate aminotransferase 1/2; ASNS = asparagine synthetase; ASS1 = argininosuccinate synthetase 1; ASL = argininosuccinate lyase; BCAT1/2 = branched-chain aminotransferase 1/2; GDH = glutamate dehydrogenase; GLS = glutaminase; GS = glutamine synthetase; OAA = oxaloacetate; PAH = phenylalanine hydroxylase; PHGDH = phosphoglycerate dehydrogenase; PSAT1 = phosphoserine aminotransferase 1; PSPH = phosphoserine phosphatase; P5CS = pyrroline-5-carboxylate reductase; PRODH = proline dehydrogenase; PYCR = pyrroline-5-carboxylate reductase; Pyr = pyruvate; 3-PG = 3-phosphoglycerate; 3-PHP = 3-phosphohydroxypyruvate; 3-PS = 3-phosphoserine; SHMT1/2 = serine hydroxymethyltransferase-1/2; THF = tetrahydrofolate; 5,10-mTHF = 5,10-methylenetetrahydrofolate; NH_3_ = ammonia.

**Figure 2:**
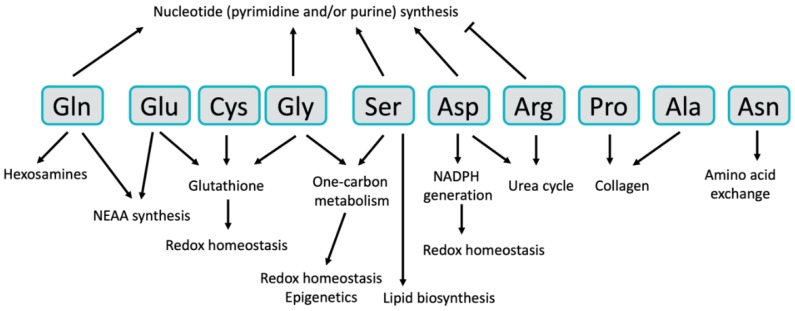
The Diverse Functional Roles of NEAA in cancer. Non-essential amino acids have diverse functions in cancer cells. NADPH = Nicotinamide adenine dinucleotide phosphate.

Glutamine is the most abundant amino acid in human plasma [15] and is one of the most studied in the context of cancer metabolism [20,21,22]. Glutamine is also the amino acid that is consumed at the highest rate by cancer cells in culture and is well-established as being required for cancer cell proliferation [5,8]. This importance is likely due to glutamine’s ability to provide both carbon and nitrogen for many biosynthetic reactions. Carbon from glutamine, in the form of α-ketoglutarate (αKG), is an important anaplerotic substrate to support the biosynthetic functions of the tricarboxylic acid (TCA) cycle [8,23], and glutamine-derived nitrogen is required for the biosynthesis of molecules such as hexosamines [9], nucleotides [10,11] and other NEAAs (Figure 1 and Figure 2). Glutamine can be taken up by cancer cells via a number of different amino acid transporters, among which ASCT2 (alanine/serine/cysteine transporter 2, coded for by the *SLC1A5* gene) is the best described [24]. Glutamine uptake is significantly increased in tumors, and glutamine-based positron emission tomography (PET) assays—similar to those currently used in the clinic for glucose—are being developed as potential clinical imaging tools [6]. Accordingly, inhibition of glutamine transporters using either small molecules or monoclonal antibodies is being explored as a potential therapeutic option [25,26,27,28]. While there is an apparent net consumption of glutamine in most cancer types, glutamine can also be synthesized from glutamate and ammonia by glutamine synthetase (coded for by the *GLUL* gene) (Figure 1), a process that is also important in cancer under some circumstances [29,30]. In addition to traditional uptake via glutamine transporters or its biosynthetic pathway, macropinocytosis and proteolytic degradation of extracellular proteins can provide an additional source of glutamine and other amino acids [31].

While numerous approaches of targeting glutamine metabolism in cancer have been proposed and tested over the last several decades [32,33,34], inhibition of glutamine catabolism by glutaminase has emerged as a major focus of both academic and pharmaceutical cancer metabolism research. Glutaminase is an enzyme that mediates the conversion of glutamine to glutamate by removing the amide nitrogen from glutamine to generate glutamate and ammonia (Figure 1). Glutaminase activity has been demonstrated to be critical for the growth of most cancer cells in culture, and several inhibitors of glutaminase have been developed [35,36,37]. The most clinically relevant glutaminase inhibitor, CB-839, has shown pre-clinical activity in a variety of mouse models and is currently in clinical trials for several tumor types [36]. While these glutaminase inhibitors are effective against most cancer cells grown in culture, often times they are less effective in mouse models of cancer [38,39]. One explanation for this *in vitro* versus *in vivo* discrepancy is the relatively high concentration of cystine in tissue culture media relative to human plasma [40]. Cystine, which is the oxidized dimer form of the NEAA cysteine (discussed in more detail below), is transported into cells in exchange for glutamate by the transporter xCT (coded for by the *SLC7A11* gene). High extracellular cystine can drive glutaminase activity by depleting the intracellular glutamate pool, thus making cancer cells more dependent on glutaminase to replenish intracellular glutamate [40]. This phenomenon also occurs in tumors with mutations in the Keap1/Nrf2 axis, as Nrf2 is the primary transcriptional driver of xCT expression [41]. These studies suggest that tumors with elevated xCT expression will be good candidates for treatment with glutaminase inhibitors. Importantly, there are additional mechanisms of resistance to glutaminase inhibition, including the ability to synthesize glutamine via glutamine synthetase [29,38,42]. Inhibition of glutaminase has also shown pre-clinical activity as part of combination therapy in several tumor types [39,43,44], further expanding the potential impact that targeting glutaminase could have on cancer treatment.

### 2.2. Glutamate

In contrast to glutamine, glutamate is not found in high concentrations in human plasma and is not typically taken up in large quantities by cancer cells. Rather, most intracellular glutamate is derived from glutamine via glutaminase (Figure 1). Glutamate can also be synthesized from branched-chain amino acids and αKG via the activity of branched-chain amino transferases (BCAT1/2), representing an important link between EAA and NEAA metabolism that is utilized in some tumors [43,45]. Glutamate occupies a central hub in NEAA metabolism, as it is important for the biosynthesis of proline, aspartate, alanine and serine, which are in turn used for the synthesis of cysteine, glycine, asparagine and arginine (Figure 1 and Figure 2). Glutamate is converted to αKG either through the action of glutamate dehydrogenase (GDH), which removes the glutamate-derived nitrogen as ammonia, or via transaminases, which transfer the nitrogen from glutamate to an α-keto acid to generate other NEAAs (Figure 1). While either route results in the generation of αKG for TCA cycle anaplerosis, the utilization of glutamate-derived nitrogen for NEAA biosynthesis may be favored in rapidly proliferating cancer cells as a mechanism of preserving nitrogen for anabolic reactions [46]. Nevertheless, inhibition of GDH, either alone or with other treatments, has been shown to inhibit tumor growth in some cancers [47,48,49,50,51], suggesting that GDH activity is important in tumors under certain circumstances. Interestingly, GDH has also been shown to operate in reverse in some breast cancer cells where it can fix nitrogen from ammonia to provide an additional source of glutamate [52]. Glutamate utilization by transaminases to generate NEAAs has also been shown to be required for tumor growth in a variety of cancer types [49,53,54,55]. Glutamate is also used for the synthesis of the antioxidant glutathione (Figure 2) [56], which is discussed in more detail in the section on cysteine. The numerous sources of glutamate available to cancer cells and the variety of pathways by which glutamate can be utilized make targeting glutamate metabolism for therapy challenging, and are an excellent example of the redundancy found in many NEAA metabolic pathways.

### 2.3. Serine

Serine is another NEAA that has garnered a great deal of attention from the cancer metabolism community. Like glutamine, serine can be taken up by numerous transporters including ASCT2 [57]. Serine is synthesized *de novo* by the serine synthesis pathway, which diverts 3-phosphoglycerate from glycolysis and utilizes nitrogen from glutamate in a three-step pathway (Figure 1). The gene for the first enzyme of the pathway, phosphoglycerate dehydrogenase (*PHGDH*), has been shown to be focally amplified in some triple-negative breast cancers and melanomas [58,59]. PHGDH and the other enzymes in the serine synthesis pathway—phosphoserine aminotransferase 1 (PSAT1) and phosphoserine phosphatase (PSPH)—can also be activated in cancer cells by epigenetic mechanisms [60] and by the transcription factor ATF4 downstream of both mTOR and Nrf2 signaling [61,62]. Serine is an important NEAA in cancer cells for several reasons, including its participation in purine biosynthesis [61,62], mitochondrial protein translation [63], lipid biosynthesis [64], and as an allosteric regulator of glycolysis [65] (Figure 2). Serine is also a critical donor of methyl groups for one-carbon metabolism, which will be discussed in the next section on glycine.

Because of its clear importance in proliferative metabolism, numerous approaches of targeting serine metabolism in tumors have been explored. Several academic laboratories and pharmaceutical companies have developed PHGDH inhibitors that have shown efficacy in some tumor models [66,67,68,69]. However, inhibition of PHGDH is not always sufficient to inhibit tumor growth [70,71], in part because serine is readily available in human plasma and can be taken up to compensate for a loss of serine biosynthesis. Interestingly, PHGDH and the serine synthesis pathway seem to be of greater importance for tumors growing in tissues that have low availability of serine in the extracellular environment [19]. In addition to inhibition of serine biosynthesis, the manipulation of serine availability by removing serine and glycine from the diet has been explored in mice as a potential therapeutic option [72,73,74]. Dietary restriction has been shown to reduce plasma serine levels by up to 75% and is effective at limiting tumor growth in a p53- and antioxidant-dependent fashion [72,73]. However, the effectiveness of dietary serine deprivation is also dependent on the ability of the tumors to synthesize serine *de novo* [19,73]. These results demonstrate a complex but important interplay between serine biosynthesis and extracellular serine availability in tumors and their environment, making it likely that identifying the appropriate approach for specific tumor types will be important if we wish to successfully target serine metabolism for cancer therapy.

### 2.4. Glycine

Serine and glycine metabolism are closely linked, as glycine is directly generated from serine via the serine hydroxymethyltransferase enzymes SHMT1 and SHMT2 (Figure 1). Importantly, the conversion of serine to glycine provides one-carbon units that are utilized by the folate and methionine cycles in the metabolic pathways collectively referred to as one-carbon metabolism. Serine, glycine, and their relation to one-carbon metabolism are highly relevant aspects of tumor metabolism that have been extensively reviewed elsewhere [75,76,77]. One-carbon metabolism is essential for the pathological functions of cancer cells for a variety of reasons, including nucleotide biosynthesis [78], nicotinamide adenine dinucleotide phosphate (NADPH) regeneration and redox homeostasis [79], protein translation [80], and epigenetic modifications [81] (Figure 2). The importance of one-carbon metabolism in cancer has been appreciated for decades. In fact, inhibition of the folate cycle was among the first effective chemotherapeutic treatments for cancer [82]. Despite these initial clinical discoveries over 70 years ago, inhibitors of folate metabolism like methotrexate are still utilized for cancer treatment today and remain an active area of research in the cancer metabolism field [75,76,77]. For example, histidine catabolism was recently shown to impact the efficacy of methotrexate treatment by reducing the cellular pool of tetrahydrofolate, suggesting that dietary histidine supplementation may improve patient response to methotrexate [83]. Not surprisingly, targeting glycine metabolism using inhibitors of the SHMT enzymes is also being explored as a potential therapeutic option [80,84,85]. Uptake of glycine from the extracellular environment [7] and the downstream utilization of glycine via the glycine cleavage system [86,87] also play important roles in cancer cells and are being investigated as potential therapeutic targets.

### 2.5. Aspartate

Numerous recent studies have demonstrated a particularly important role for aspartate metabolism in cellular proliferation and cancer. Aspartate is generated from oxaloacetate and glutamate-derived nitrogen by aspartate aminotransferase enzymes (Figure 1), of which there are cytosolic and mitochondrial isoforms (coded for by the *GOT1* and *GOT2* genes, respectively). The role of aspartate in transferring electrons between the cytosol and mitochondria via the malate–aspartate shuttle is well understood and as such it is believed that the majority of aspartate synthesis in rapidly proliferating cells occurs in the mitochondria [88]. Indeed, transport of aspartate from the mitochondria to the cytosol via the aspartate–glutamate carrier is important for cell survival under certain conditions [89]. As mentioned, the concentration of aspartate in plasma is the lowest among the proteinogenic amino acids [15] and aspartate is not efficiently transported into most cancer cells [88], suggesting that biosynthesis via aspartate aminotransferase is the most relevant source of aspartate in most cancer cells. Aspartate is essential for the synthesis of both purine and pyrimidine nucleotides (Figure 2), and as such aspartate synthesis is very closely linked to cellular proliferation [46]. Aspartate metabolism can also be an important source of NADPH utilized for the neutralization of reactive oxygen species in certain cell types, thereby promoting biosynthesis and cellular survival [49].

Several recent reports have uncovered an interesting connection between the mitochondrial electron transport chain and aspartate biosynthesis. These studies have suggested that the essential function of the mitochondrial electron transport chain in proliferating cells is to facilitate aspartate biosynthesis [88,90]. In this model, the electron transport chain serves as an electron acceptor, consuming nicotinamide adenine dinucleotide (NADH) to regenerate NAD^+^, which can then be utilized for oxaloacetate generation and aspartate biosynthesis. Indeed, provision of exogenous aspartate is sufficient to rescue electron transport chain deficiency in cancer cells [88,90]. This result is remarkable given the numerous other functions of mitochondrial oxidative phosphorylation but, nevertheless, stresses the importance of aspartate biosynthesis in proliferative cells. Importantly, aspartate availability has recently been shown to be limiting for tumor growth *in vivo* [91], and inhibition of aspartate biosynthesis can inhibit tumor growth [49,53]. These studies demonstrate the utmost importance of aspartate in cancer and have motivated the development of inhibitors of aspartate aminotransferases as potential cancer therapeutics [92,93].

### 2.6. Asparagine

Asparaginase, an injectable enzymatic drug that degrades asparagine in the plasma, is a “cornerstone” of treatment for acute lymphoblastic leukemia (ALL) [94]. Thus, asparaginase is likely the most prominent example of a current cancer therapy that directly targets NEAA metabolism. Acute lymphoblastic leukemia cells are sensitive to the depletion of asparagine in the plasma in part because they lack significant expression of asparagine synthetase (coded for by the *ASNS* gene) [94,95], the enzyme that synthesizes asparagine using aspartate and nitrogen from glutamine (Figure 1). This results in a severe lack of asparagine for protein synthesis in ALL cells and subsequent induction of apoptosis. Resistance to asparaginase treatment can occur in ALL and is commonly caused by induction of asparagine synthetase expression and a renewed ability to synthesize asparagine [95]. Accordingly, asparagine synthetase inhibitors have been developed and can overcome resistance to asparaginase treatment [96,97]. While the clinical utility of asparaginase makes it clear that asparagine is essential for tumor growth, the importance of asparagine beyond protein synthesis is less understood. However, asparagine has been shown to function as an important exchange factor needed for the uptake of other amino acids that are required for the activation of mTOR signaling (Figure 2) [98]. This suggests a potential feedback mechanism where low asparagine levels can be sensed by mTOR signaling to reduce the rates of protein synthesis. Interestingly, intracellular asparagine levels have recently been shown to be required for breast cancer metastasis [99], suggesting that asparaginase treatment, dietary asparagine limitation, or inhibition of asparagine synthetase may be effective treatment options for metastatic breast cancer.

### 2.7. Alanine

Alanine lies at a central hub of carbon metabolism, being synthesized by alanine aminotransferases (coded for by the *GPT* and *GPT2* genes) using carbon from pyruvate and nitrogen from glutamate (Figure 1). Despite these connections to highly cancer-relevant metabolic pathways, the role of alanine in cancer is less understood relative to some other NEAAs. It is interesting to speculate that this may be in part because of a discrepancy between the concentration of alanine in human plasma, where it is the second most abundant amino acid, and in most tissue culture medias, which have little to no alanine [15]. This forces cancer cells in culture to synthesize nearly all of their alanine regardless of whether this would normally occur in a tumor, and has the potential to lead to tissue culture-generated artifacts. This stresses the potential importance of using tissue culture media that more accurately represent the nutrient levels found *in vivo* [15,29,40,100]. Despite these inconsistencies, there is some emerging evidence of the importance of alanine metabolism in cancer. For example, biosynthesis of alanine has been shown to be correlated with proliferation, suggesting that it may play a role in proliferative cell metabolism [46]. Alanine is also an important survival signal in pancreatic cancer, where stromal cells promote the proliferation and survival of pancreatic cancer cells by secreting alanine that can be utilized in the TCA cycle of the cancer cells [101]. In addition, a recent report has demonstrated that alanine aminotransferase is an important source of αKG for the hydroxylation of collagen and the preparation of the metastatic niche in breast cancer (Figure 2) [102]. These studies suggest that alanine indeed plays an important role in cancer biology, but additional work will likely be needed to motivate the development of alanine-targeted therapies.

### 2.8. Cysteine

One of two sulfur-containing proteinogenic amino acids, cysteine is unique in that it contains a reactive thiol side chain that endows several functions not possible with other amino acids. For example, reactive cysteine residues are often found in the catalytic site of enzymes where they function as a nucleophile in enzyme-catalyzed reactions [103]. Cysteine also forms disulfide bonds with other cysteines, a function that is critical in promoting protein folding and stability [104]. Reactive cysteine residues are also the driving force behind the ability of antioxidants to quench reactive oxygen species [104,105]. These diverse functional roles have made cysteine one of the more heavily studied NEAAs in cancer. Cysteine can be synthesized *de novo* from serine and methionine in a pathway known as the transsulfuration pathway (Figure 1). While this pathway has been shown to contribute to cysteine production in cancer cells under certain circumstances [106,107,108], the majority of intracellular cysteine is taken up from the extracellular environment either as cysteine [109] or in its oxidized dimer form, cystine. Cystine is transported into cells via the transporter xCT and then reduced to cysteine by thioredoxin reductase 1 and glutathione reductase [110]. Cystine uptake plays an important role in cancer, as evidenced by numerous attempts at inhibiting xCT as a potential therapeutic target in cancer [111,112,113,114]. Interestingly, inhibition of cystine uptake induces a unique form of cell death known as ferroptosis [115], the molecular components of which are also being tested as potential cancer therapeutics [116,117,118]. Enzymatic depletion of plasma cystine and cysteine—similar to the approach used for asparagine with asparaginase—can also suppress tumor growth [119]. Importantly, the predominant transcriptional regulator of cysteine metabolism—the Keap1/Nrf2 pathway—is mutated in numerous tumor types [120,121,122] and can be activated by oncogenic signaling pathways such as KRas and PI3K [123,124], suggesting that downstream control of cysteine metabolism is an important oncogenic function.

As mentioned, one of the key functions of cysteine in cancer is its role in reactive oxygen defense as part of several antioxidant systems (Figure 2) [105]. Of particular relevance for this review is the metabolite antioxidant glutathione, which is a tripeptide synthesized from three NEAAs—cysteine, glutamate and glycine [56]. The potential importance of glutathione metabolism in cancer is evidenced by the observation that glutathione is one of the most significantly increased metabolites in tumors relative to normal tissue [125,126]. Further, glutathione biosynthesis is required for tumor initiation and progression [113], and numerous oncogenic alterations promote glutathione biosynthesis by activating the Keap1/Nrf2 pathway [114,123,124]. Interestingly, despite being one of the most abundant metabolites in cancer cells, many cancer cells are resistant to inhibition of glutathione biosynthesis [113,127], indicative of functional redundancy in cellular antioxidant systems. However, targeting glutathione biosynthesis as part of combination therapy is effective in many circumstances [43,113,127], suggesting a potential role for the inhibition of NEAA utilization for glutathione biosynthesis as a therapeutic strategy.

### 2.9. Arginine

Arginine is a component of the urea cycle, a metabolic pathway that converts the toxic metabolic byproduct ammonia to urea to be excreted in urine (Figure 2). This process takes place primarily in the liver, and it has been observed that the urea cycle is suppressed in many tumors [128]. Mechanistically, suppression of the urea cycle in tumors is often accomplished through the epigenetic silencing of two urea cycle genes, *ASS1* and *ASL* [129,130,131]. *ASS1* and *ASL* suppression in tumors is believed to be beneficial for tumor growth because it diverts nitrogen into aspartate for pyrimidine biosynthesis [132]. While beneficial for promoting anabolic metabolism, the suppression of the urea cycle prevents these tumors from synthesizing arginine *de novo*, making them dependent on the uptake of arginine from the circulation (Figure 1) [133,134,135]. This makes these tumors sensitive to the enzymatic depletion of plasma arginine, an approach that has been explored in clinical trials as a therapeutic option [136,137,138,139]. Interestingly, while the elevated pyrimidine biosynthesis found in urea cycle-deficient tumors promotes proliferation, it also causes an imbalance in purine and pyrimidine nucleotide levels that leads to an increased mutational load [140]. This increased mutational load in turn increases the immunogenicity of these tumors and increases their responsiveness to checkpoint inhibitors [140]. Therefore, the alterations in arginine metabolism in cancer are an excellent example of how metabolic changes that support tumor growth can induce collateral vulnerabilities that can potentially be taken advantage of for cancer therapy.

### 2.10. Proline

Proline is unique among the proteinogenic amino acids for its cyclic shape, which allows for variability in protein structure. This is particularly important in proteins such as collagen, which contains a large amount of proline and is important for the structural elements of the extracellular matrix (Figure 2) [141]. Proline can be synthesized from glutamate through the action of P5C synthase and pyrroline-5-carboxylate reductase and can be degraded by proline dehydrogenase (also known as proline oxidase) (Figure 1). Both the biosynthetic and degradation pathways for proline can be regulated by MYC, demonstrating that proline metabolism is also altered by oncogenic signaling pathways [142]. Interestingly, proline catabolism through proline dehydrogenase has been shown to promote cancer cell survival [143] and metastasis [144] but may also have a tumor-suppressive function [145]. These contrasting roles suggest a context-dependent role of proline metabolism in cancer. Proline metabolism in tumors is also important for bioenergetics, osmoregulation, stress protection, and control of apoptosis [146]. In addition to the biosynthetic pathway and uptake of proline, degradation of collagen in the extracellular matrix through macropinocytosis can provide an additional source of proline to pancreatic cancer cells under metabolic stress [147]. Despite these redundant sources of proline available to tumors, ribosome profiling studies have suggested that proline levels are limiting for protein synthesis in some tumors [148]. This suggests that targeting proline metabolism may be a viable therapeutic option for cancer treatment.

### 2.11. Tyrosine

Unlike the other NEAAs that reside in an interconnected network of metabolic pathways, tyrosine is synthesized from the EAA phenylalanine via phenylalanine hydroxylase (coded for by the *PAH* gene) (Figure 1). As mentioned, tyrosine is non-essential but can become essential in phenylketonuria, which is caused by mutations in the *PAH* gene [14]. While relatively little is known about the biology of tyrosine in cancer beyond its importance for protein synthesis, there have been efforts to take advantage of tyrosine metabolism in the clinic. For example, tyrosine-based PET imaging techniques have been developed and can be effective at imaging tumors and therapeutic responses [149,150,151]. It is believed that tyrosine PET tracers are effectively a readout of the activity of the amino acid transporter LAT1 [152,153], the expression and activity of which is elevated in numerous tumor types [154,155]. Interestingly, there is also a tyrosine-mimetic drug, SM-88, that is under development for the treatment of several cancer types and is in active clinical trials [156]. While details of SM-88 are limited, it will be interesting to see the results of these trials and learn more about this effort to target tyrosine metabolism for therapy.

## 3. Conclusions

Years of research have provided an increasingly clear picture of the diverse and important roles that NEAAs play in tumor metabolism. In addition to initial studies emphasizing the role of glutamine and glutamate metabolism in cancer, there is continually accumulating evidence that other NEAAs also play critical roles in the pathology of cancer. Importantly, several novel strategies to target NEAA metabolism are currently in clinical trials. In addition, dietary manipulation of NEAA metabolism is also a potentially effective strategy for inhibiting tumor growth that is being explored either as a primary treatment option or to enhance the efficacy of other chemotherapies. Importantly, the manipulation of dietary amino acid levels can have adverse effects on normal physiology [157], suggesting that strategies to manipulate dietary NEAAs will need to be developed carefully in order to avoid negative side effects. In addition, the inherent redundancy of NEAA metabolic pathways and the multiple sources of NEAAs available to cancer cells all combine to make targeting NEAA metabolism challenging. In the most successful examples of targeting NEAA metabolism for therapy, such as in ALL which lacks the ability to synthesize asparagine and is sensitive to asparaginase treatment, identifying circumstances where NEAA pathway redundancy is naturally limited can improve the likelihood of successfully targeting these pathways for therapy. As more NEAA-targeted therapies move towards the clinic, it will be exciting to observe the creative approaches that researchers develop to overcome these challenges.
